# The academic outcomes of working memory and metacognitive strategy training in children: A double‐blind randomized controlled trial

**DOI:** 10.1111/desc.12870

**Published:** 2019-06-27

**Authors:** Jonathan S. Jones, Fraser Milton, Mohammod Mostazir, Anna R. Adlam

**Affiliations:** ^1^ School of Psychology, College of Life and Environmental Sciences University of Exeter Exeter UK; ^2^ MRC Cognition and Brain Sciences Unit University of Cambridge Cambridge UK

**Keywords:** children, mathematics, metacognitive strategy, reading, working memory, working memory training

## Abstract

Working memory training has been shown to improve performance on untrained working memory tasks in typically developing children, at least when compared to non‐adaptive training; however, there is little evidence that it improves academic outcomes. The lack of transfer to academic outcomes may be because children are only learning skills and strategies in a very narrow context, which they are unable to apply to other tasks. Metacognitive strategy interventions, which promote metacognitive awareness and teach children general strategies that can be used on a variety of tasks, may be a crucial missing link in this regard. In this double‐blind randomized controlled trial, 95 typically developing children aged 9–14 years were allocated to three cognitive training programmes that were conducted daily after‐school. One group received Cogmed working memory training, another group received concurrent Cogmed and metacognitive strategy training, and the control group received adaptive visual search training, which better controls for expectancy and motivation than non‐adaptive training. Children were assessed on four working memory tasks, reading comprehension, and mathematical reasoning before, immediately after, and 3 months after training. Working memory training improved working memory and mathematical reasoning relative to the control group. The improvements in working memory were maintained 3 months later, and these were significantly greater for the group that received metacognitive strategy training, compared to working memory training alone. Working memory training is a potentially effective educational intervention when provided in addition to school; however, future research will need to investigate ways to maintain academic improvements long term and to optimize metacognitive strategy training to promote far‐transfer. A video abstract of this article can be viewed at https://youtu.be/-7MML48ZFgw

## Graphical abstract

Working memory training in schools was found to improve children's working memory performance and mathematical reasoning ability. Working memory benefits were maintained three months later and were significantly greater for children that received additional metacognitive strategy training.

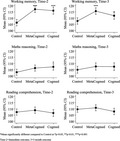

**Research Highlights**

- Ninety‐five children were randomly allocated to receive working memory training, working memory and metacognitive strategy training, or adaptive visual search training (control group) after‐school.
- Working memory performance significantly improved in both training groups relative to the adaptive control group and was maintained 3 months later.
- Improvements in maths were also observed immediately after training, suggesting that working memory training may improve children’s academic outcomes when provided in addition to school.
- The addition of metacognitive strategy training was associated with greater improvements in working memory performance at 3 months, compared to working memory training alone.

## 1 INTRODUCTION

Working memory is a promising target for cognitive training because it is related to a wide range of cognitive functions (Barrett, Tugade, & Engle, 2004). It is a system for retaining and manipulating information over a few seconds (Baddeley & Hitch, 1994), which is involved in reading (Daneman & Carpenter, 1980), mathematics (Peng et al., 2015) and reasoning (Kane et al., 2004). Furthermore, studies have shown that working memory capacity predicts children's academic achievement (Alloway & Alloway, 2010; Gathercole, Pickering, Knight, & Stegmann, 2004), and those that leave school with better qualifications have higher rates of employment and income (Office for National Statistics, 2013). Thus, if working memory capacity can be increased through training, it could have far‐reaching impact by improving children's cognitive abilities, academic achievement and future life outcomes.

Working memory training programmes, such as Cogmed (Klingberg et al., 2005), n‐back and dual n‐back training (Jaeggi, Buschkuehl, Jonides, & Perrig, 2008; Jaeggi et al., 2010), simple‐ and complex‐span training (Harrison et al., 2013), and binding training (De Simoni & von Bastian, 2018), typically involve intensive and prolonged practice on one or multiple tasks that adapt in difficulty. A recent meta‐analysis of these programmes in typically developing children (including 26 studies and 1,601 children aged 3–16 years) showed significant improvements on untrained working memory tasks, that is near‐transfer, which were maintained 3–6 months later (Sala & Gobet, 2017). Regarding far‐transfer to other domains, significant immediate improvements were reported for mathematics, but not reading, science, fluid intelligence, crystallized intelligence or cognitive control. However, interpretation of these findings is limited because the analyses included studies with passive, that is no intervention, control groups (e.g., Witt, 2011), which do not control for possible placebo, Hawthorne, or demand effects (Shipstead, Redick, & Engle, 2010) or non‐specific effects of the training on children's motivation and attentional capacity (Shipstead, Hicks, & Engle, 2012). In order to make strong inferences regarding the effectiveness of working memory training, it is, therefore, essential that an active control group is employed that will engage children in some form of training, which is similar in terms of expectancy, motivation and demands on sustained attention.

When only considering studies with active control groups, Sala and Gobet's (2017) meta‐analysis revealed a significant near‐transfer effect in the short term and no evidence of far‐transfer effects. No such analysis was performed for long‐term near‐transfer due to a paucity of studies, which have currently provided mixed evidence (Henry, Messer, & Nash, 2014; Hitchcock & Westwell, 2017; Karbach, Strobach, & Schubert, 2015; Studer‐Luethi, Bauer, & Perrig, 2016). Another meta‐analysis showed that working memory training may improve self‐ or parent‐reported inattention symptoms (Spencer‐Smith & Klingberg, 2015). In typically developing children, training has been found to improve maths ability compared to education as usual (St Clair‐Thompson et al., 2010; Witt, 2011), but not when compared to an active control (Henry et al., 2014). Similarly, Cogmed has been associated with improvements in maths and reading in typically developing children when compared to education as usual (Söderqvist & Bergman‐Nutley, 2015). However, the only randomized controlled trial of Cogmed in typically developing children to date found no evidence of far‐transfer to mathematics or reading comprehension compared to non‐adaptive Cogmed, where the difficulty of training remained at a low level (Hitchcock & Westwell, 2017). Overall, these findings suggest that training can improve working memory; however, it is unclear if these effects are maintained long term and there is little evidence of far‐transfer to other domains, when expectancy and motivation are appropriately controlled for.

One possible reason for the lack of far‐transfer effects in studies with active control groups may be because working memory training primarily promotes the acquisition of highly task‐specific strategies (Dunning & Holmes, 2014; Randall & Tyldesley, 2016). For example, Cogmed has been shown to increase the use of grouping strategies and performance on near‐transfer tasks that are structurally similar to the training tasks (Dunning & Holmes, 2014), but not on structurally different tasks such as the n‐back (Ang, Lee, Cheam, Poon, & Koh, 2015). Far‐transfer may be limited because working memory strategies do not afford performance on other tasks, such as reading comprehension (Bailey, Dunlosky, & Kane, 2008; Dunlosky & Kane, 2007) or reasoning (Bailey, Dunlosky, & Kane, 2011; Dunlosky & Kane, 2007).

Alternatively, working memory training may primarily increase capacity (Klingberg, 2010; Lövdén et al., 2010), but children may be unable to intuitively apply this to other cognitive or academic tasks. One theory suggests that high working memory capacity affords the production and implementation of effortful and effective strategies on cognitively demanding tasks (Dunlosky & Kane, 2007). However, children may require specific instruction and guidance on how to apply their newly acquired additional capacity in other situations (Partanen, Jansson, Lisspers, & Sundin, 2015), particularly as children's ability to generate and utilize strategies is still developing (Bjorklund, Dukes, & Brown, 2008). Far‐transfer may depend upon metacognition to mindfully abstract something learnt in one context and apply it to a new context (Salomon & Perkins, 1989). Self‐regulated learning is thought to be achieved through planning, monitoring, and evaluating (Pintrich, 2000; Zimmerman, 2002), and these domain‐general strategies are typically taught in metacognitive strategy interventions (Fisher, 1998), which are recommended as one of the most impactful educational interventions (Higgins et al., 2016). Such interventions have been shown to improve children's academic performance (Dignath & Büttner, 2008) and generalize to attainment in untrained school subjects, compared to education as usual (Adey & Shayer, 1993). Concurrent working memory and metacognitive training may, therefore, facilitate children's performance across a range of tasks by increasing cognitive capacity, metacognitive awareness and utilization of general strategies.

The academic outcomes of Cogmed with metacognitive strategy training have previously been investigated in children with special educational needs, in comparison with Cogmed alone and education as usual (Partanen et al., 2015). Metacognitive strategy training was provided in three additional sessions each week that focused on labelling elements of the training tasks, formulating goals, identifying strategies and pitfalls, sharing planning and execution strategies, and relating the training tasks to school or leisure time. Cogmed and metacognitive strategy training was found to improve visuospatial working memory, which was maintained 6 months later; but this was only in comparison with education as usual and there was no evidence of far‐transfer to mental arithmetic, reading comprehension, writing or nonverbal reasoning. No improvements were observed in the group that received Cogmed alone, which led to the suggestion that training children's metacognitive skills and strategies might be an important prerequisite for transfer in children with special educational needs. However, this effect was confounded by the additional contact time that the metacognitive group received and, as discussed earlier, improvements relative to a passive control group should be treated with caution.

The academic outcomes of working memory and metacognitive strategy training in typically developing children have been more promising, but only one study has been conducted to date (Carretti, Caldarola, Tencati, & Cornoldi, 2014). Children practised on three working memory tasks of increasing difficulty and were taught metacognitive strategies in the context of reading comprehension in 22 biweekly sessions, each lasting 1 hr. Specifically, children were taught how to identify goals, monitor their comprehension and predict the content of the reading based on the genre. In addition, children were taught reading strategies and how to integrate information between texts and pictures. Compared to controls who practised reading comprehension exercises for the same amount of time, the intervention was found to significantly improve working memory and reading comprehension. This suggests that concurrent working memory and metacognitive training may improve academic outcomes. However, it is unclear whether the improvement in reading comprehension was an effect of working memory training, metacognitive strategy training, instruction in specific reading strategies, training in integration skills or a combination of these. There is extensive evidence that instruction in reading strategies improves children's comprehension (Higgins et al., 2016); however, it is unlikely that these benefits would generalize further (Lustig, Shah, Seidler, & Reuter‐Lorenz, 2009). It is, therefore, important to determine the specific effects of metacognitive strategy training in addition to working memory training, as this may be crucial to promote far‐transfer to academic outcomes.

### 1.1 Adaptive control groups

As mentioned previously, a common discussion in the cognitive training literature concerns what constitutes an appropriate control group (Simons et al., 2016). Although most recent studies have used active rather than passive control groups, the vast majority have used a non‐adaptive variant of the training programme, where the difficulty remains at a low level and users receive no feedback. This may not sufficiently control for expectancy and motivation because users receiving adaptive training are constantly challenged and receive feedback on their improvements, which may bias their performance at outcome (Shipstead et al., 2012). Many recent reviews have consequently recommended using an adaptive control group that effectively trains a capacity unrelated to working memory (Boot, Simons, Stothart, & Stutts, 2013; Green, Strobach, & Schubert, 2014; Noack, Lovden, & Schmiedek, 2014; Shipstead et al., 2012; Simons et al., 2016). Individuals in an adaptive control group should be challenged, see improvements during training, and, therefore, have similar expectancy and motivation at assessment.

Only one published investigation of working memory training in children has used an adaptive control group (Jaeggi, Buschkuehl, Jonides, & Shah, 2011). The control group received adaptive knowledge training, which involved answering questions in a multiple‐choice quiz format. Each training session consisted of 10 one‐minute blocks that adapted in difficulty by presenting more difficult questions when there were fewer than four errors and easier questions when there were four or more errors. Furthermore, new or previously incorrectly answered questions were presented at each session. Only children who made large improvements on the n‐back training task showed improvements in fluid intelligence relative to the control group. Whilst these results are intriguing, there was no direct measure of working memory performance or far‐transfer to academic outcomes. Furthermore, the difficulty of questions was somewhat subjective and the training did not place demands on visuospatial attention, which is typically controlled for in non‐adaptive training (Klingberg et al., 2005). Adaptive visual search training has been used as a control group in multiple working memory training studies in adults (Covey, Shucard, & Shucard, 2019; Harrison et al., 2013; Redick et al., 2013) but not yet in children. This approach may be a more suitable control for the visuospatial and attentional demands of working memory training in children. In addition, the difficulty of the task adapts in a more objective manner, by increasing or decreasing the number of distracters in the search array according to performance.

### 1.2 The present study

In summary, whilst there is some suggestive evidence that working memory training can improve children's working memory performance, the limitations of the control tasks used in most of these studies mean that such a conclusion needs to be taken with caution. Furthermore, there is currently very little evidence indicating that working memory training, on its own, can improve children's academic outcomes. Our review of the literature has indicated that there is not only a clear need for more developmental studies utilizing effective adaptive control tasks, but also that a priority should be to explore whether combining working memory training with other training methods is more effective than working memory training alone. For this latter issue, there appear to be compelling theoretical reasons why metacognitive training may facilitate far‐transfer of working memory training and there is some preliminary support from a single study that this is indeed the case (Carretti et al., 2014). However, whilst Carretti et al.’s study makes a valuable contribution to the field, the inferences that can be made about what is driving their observed effect are limited. Further investigation is necessary to determine whether there is a sound scientific basis for the widespread use of working memory training regimes within schools and at home. The present study aims to provide an important next step in addressing these outstanding issues, the resolution of which would not only have important theoretical implications but could also have a substantial beneficial impact on future educational training programmes.

To this end, the present study aimed to: (a) investigate the immediate and 3‐month outcomes of working memory training in typically developing children when compared to an adaptive control group; and (b) investigate whether combined working memory and metacognitive strategy training facilitates far‐transfer to academic outcomes compared to working memory training alone and an adaptive control group. To address these aims, a double‐blind randomized controlled trial was conducted with typically developing children (aged 9–14 years) who were randomly assigned to receive either Cogmed, Cogmed and metacognitive strategy training (“MetaCogmed”), or adaptive visual search training (Control). The primary outcomes were average performance on four working memory tasks, mathematical reasoning and reading comprehension. We also examined the extent of near‐transfer to the individual working memory tasks in consideration of the similarities and differences to the Cogmed training tasks.

Cogmed was selected as a suitable working memory training programme because it is widely used and has been associated with large near‐transfer effects in typically developing children (Astle, Barnes, Baker, Colclough, & Woolrich, 2015) and across populations (Melby‐Lervåg & Hulme, 2013). We note that Cogmed includes training on both short‐term memory and working memory tasks, that is tasks that just require storage and tasks that require processing and storage. Accordingly, we measured near‐transfer to short term and working memory tasks. For the purposes of this investigation, we treat short‐term memory as a facet of working memory, as is the case in some models (e.g., Baddeley & Hitch, 1974). The MetaCogmed group received the standard Cogmed protocol combined with a novel metacognitive strategy workbook developed from the education literature (Fisher, 1998). The workbook taught children how to plan, monitor and evaluate whilst completing the Cogmed training tasks, reading comprehension exercises and word‐based maths problems. Children in the other two training groups received placebo workbooks without the metacognitive content. All training was provided after‐school, as previous evidence indicates that training in lieu of school lessons is detrimental to long‐term academic outcomes (Roberts et al., 2016). A 3‐month follow‐up was included to examine long‐term academic outcomes and whether near‐transfer effects are maintained, as previous studies have provided mixed evidence of long‐term near‐transfer.

## 2 METHOD

### 2.1 Participants

**Figure 1:**
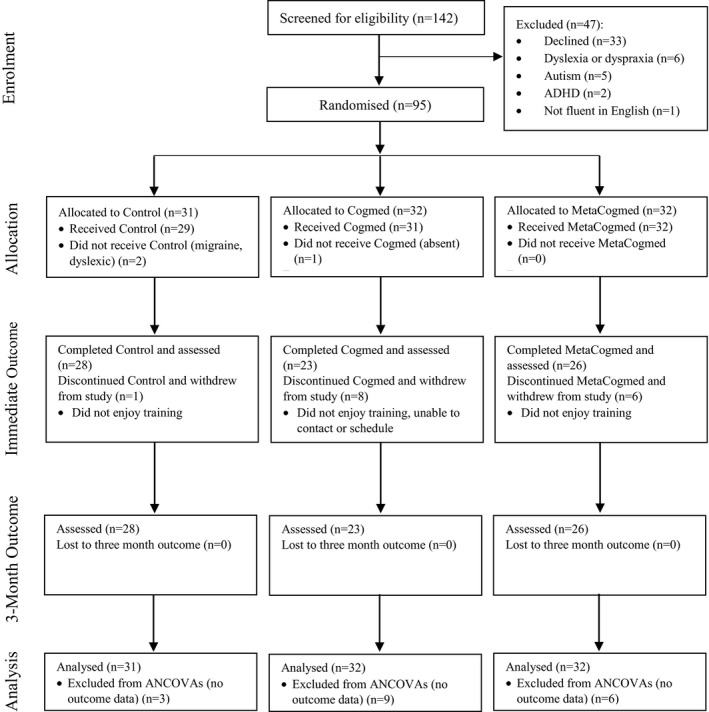
CONSORT flow diagram. Abbreviation: ANCOVAs, analyses of covariance

Initially, 142 parents/guardians expressed interest in their children taking part in the study. After exclusions and withdrawals of interest (see Figure 1 for participant flow), 95 typically developing children aged 9–14 years (*M* = 12.51, *SD* = 1.18) were recruited from four schools in Devon, England between September 2016 and June 2017. The sample included 45 girls (47.4%) and 50 boys (52.6%), who were primarily white British. Seven children were recruited from one primary school and 88 children from three secondary schools. Children were excluded if they had a diagnosis of a developmental disorder, acquired brain injury, or uncorrected visual, hearing or motor impairment that might hinder their engagement with the training. All participating children provided written assent and their parent/guardian provided written consent. The study was approved by the University of Exeter Ethics Committee (Ref: 2016/1288).

### 2.2 Design

This study utilized a double‐blind randomized controlled trial where the intervention and data collection were conducted in participating schools. Participants in each school were randomly allocated 1:1:1 to three training conditions: Cogmed, MetaCogmed and Control. Randomization was completed by researchers who were not involved in conducting outcome assessments at that particular school. Numbers were randomly generated on a computer, and repeated until group sizes were equal or differed by one.

### 2.3 Procedure

Children and parents/guardians were informed that the aim of the study was to compare two computerized cognitive training programmes and two workbooks, which would be randomly assigned to participating children. The MetaCogmed and Cogmed groups completed the Cogmed RoboMemo programme (see https://www.cogmed.com/rm for full details) and the Control group received adaptive visual search training. In addition, the MetaCogmed group received a metacognitive workbook and the Cogmed and Control groups received a placebo workbook. All materials and data are publicly available (https://osf.io/kxyf3). The training was conducted as an afterschool club, where children trained together in one of the school's computer rooms for approximately 1 hr following the end of the normal school day. The afterschool club ran every day for 6–7 weeks, and all training groups were instructed to complete 20–25 training sessions in that time, in accordance with the Cogmed protocol. The sessions were supervised by one to three members of the research team who were certified Cogmed coaches. The coaches provided technical support, clarification and encouragement to all participating children who otherwise trained independently. Parents and guardians were contacted weekly with updates on their child's progress and any difficulties with their child's compliance to the training protocol were discussed. Children were rewarded with a £1 Amazon voucher or item of stationery every time they completed four training sessions, and a £15 Amazon voucher when they completed 20 training sessions. At the end of the study, children who were assigned to the control group were offered a free license to use Cogmed in their own time.

Standardized assessments were administered by trained researchers who were unaware of children's group assignment. Baseline assessments of working memory, IQ, reading comprehension and mathematical reasoning were conducted at each school 0–21 days before training (*M* = 7.59, *SD* = 5.41). Children were reassessed on the working memory, reading comprehension and mathematical reasoning tasks 1–17 days after completing the training programme (*M* = 3.87, *SD* = 3.63) and again at least 3 months later (*M* = 14.42 weeks after training, *SD* = 1.06).

#### 2.3.1 Cogmed working memory training

Cogmed RoboMemo included a battery of 11 short‐term and working memory tasks with visuospatial and verbal stimuli (Klingberg et al., 2005). The tasks required recalling a sequence of spatial locations in order, tracking and recalling a sequence of moving spatial locations or objects, reordering and recalling a sequence of spatial locations, recalling a sequence of digits in reverse order and recognizing a sequence of letters. Each session lasted approximately 45 min with breaks and involved training on eight tasks for approximately 15 trials each. The difficulty of the training tasks adapted on a trial‐by‐trial basis according to the individual's performance. The complexity of the stimulus sequence or the number of items to remember would increase following successful responses, whereas they would decrease following incorrect responses. To control for time spent training, the number of trials adapted to an individual's processing time and current span level, where fewer trials were presented for children training at a higher span and more trials were presented for children training at a lower span. Cogmed included a difficulty level meter, high scores, audio and verbal feedback, and a reward game “Robo Racing.” At the end of each session, performance was converted into lives that children could use to play Robo Racing for a few minutes, depending on how many lives they had accrued during training. Children used the arrow keys to move a robot left, right, and up in the air to collect stars and race against the clock.

#### 2.3.2 Adaptive visual search training

Adaptive visual search training has recently been shown to be an excellent control for subjective expectations and is unlikely to produce transfer effects because performance is only very weakly correlated with working memory and other executive functions (De Simoni & von Bastian, 2018). We developed an adaptive visual search training programme in OpenSesame 3.1 (Mathôt, Schreij, & Theeuwes, 2012) called “Codebreak,” which was based on a previous paradigm (Redick et al., 2013). A narrative, colour scheme, motivational feedback and high scores were added to make this more engaging for children. The training involved adaptive practice on a visual search task where each session consisted of 24 blocks of 24 trials, lasting approximately 45 min with breaks. Children searched for the target letter “F” amongst an array of distracter letters which consisted of ‘E's or ‘t's. The letters could either face to the right, as normal, or to the left, as mirror images. Each trial began with a fixation dot presented for 500 ms, followed by an array for 500 ms, and then a mask for 2,500 ms. Children had to report the orientation of the target by pressing the right arrow when the “F” was facing to the right, or left arrow when it was facing to the left. Feedback was given on each trial in the form of a tone presented for 200 ms; a high tone indicated a correct response and a low tone indicated an incorrect response. If no response was made during the presentation of the array or mask, the trial was considered incorrect.

Similar to Cogmed, the visual search training adapted in difficulty and provided feedback on performance. The search array began at 2 × 2 and adapted at the end of each block. If accuracy was >87.5% the difficulty was increased by adding a row or column to the array, if accuracy was between 75% and 87.5% the difficulty remained the same, and if accuracy was <75% the difficulty was reduced by removing a row or column from the array. A difficulty level of one indicated a 2 × 1 array, a difficulty level of two indicated a 2 × 2 array, a difficulty level of three indicated a 3 × 2 array, and so on. Verbal and audio feedback was provided at the end of each block and each session. As in Cogmed, children would start a new session at the same difficulty level they had ended on in the previous session.

#### 2.3.3 Metacognitive workbook

The metacognitive workbook trained children on three strategies: planning, monitoring and evaluating, which are commonly used in educational interventions (Fisher, 1998). Children first completed three reflection exercises, which encouraged them to think about their thinking as they completed a Cogmed training task, a reading comprehension exercise, and a word‐based maths problem. They were then introduced to planning, monitoring, evaluating and specific metacognitive strategies that serve to self‐motivate and refocus. The motivation and focus strategies were adopted from paediatric neurorehabilitation programmes that combine training of attention and memory with instruction in metacognitive strategies (Butler & Copeland, 2002; Sohlberg, Harn, MacPherson, & Wade, 2014). Whilst completing the Cogmed training tasks, reading comprehension exercises, and word‐based maths problems, questions prompted children to plan before starting the task, reminded them to monitor their thoughts during the task and required them to evaluate their thinking after the task. The questions particularly focused on the goal of the task itself, generation of task‐specific strategies which might aid performance, steps needed to complete the task and strategies to improve motivation and focus. As children progressed through the workbook, the questions were replaced with prompts to encourage children to remember how to plan, monitor and evaluate. Children were not instructed to use any task‐specific mnemonic, reading, mathematical or problem‐solving strategies, but were instead encouraged to generate and implement their own cognitive strategies. In addition, the children wrote down how to use, when to use and why to use these strategies in their “Personal Strategy Guide” (Schraw, 1998), which was available throughout training.

Children in the Cogmed and Codebreak groups received a placebo workbook, which was designed to have face validity and to hold children's attention for a similar amount of time to the metacognitive workbook. In place of the metacognitive content, the placebo workbook contained word searches that were related to the readings, number searches linked to the maths problems and questions pertaining to the acceptability of the training.

The metacognitive and placebo workbooks consisted of written information, illustrations and exercises, including six reading comprehension exercises and six word‐based maths problems. The workbooks were divided into 25 sections that each took 10–15 min to complete. Children independently completed one section after each session of their computerized training. The coaches checked the workbooks during and after each session to ensure that children had completed the appropriate section and in sufficient detail. To ensure that the language and difficulty of the exercises were age‐appropriate, two versions of the workbooks were developed. One was designed for primary school children aged 9–11 years and the other for secondary school children aged 11–14 years.

### 2.4 Measures

IQ was assessed to characterize the sample at baseline using the two sub‐tests version of the Wechsler Abbreviated Scale of Intelligence–II (WASI‐II; Wechsler, 2011). This includes a measure of crystallized intelligence (Vocabulary) and a measure of fluid intelligence (Matrix Reasoning). The WASI‐II has excellent internal consistency (α = 0.93), test–retest reliability (*r* = 0.87–95) and inter‐rater reliability (McCrimmon & Smith, 2013).

Working memory was assessed at baseline, immediate outcome, and the 3‐month outcome using four tasks from the Automated Working Memory Assessment (AWMA; Alloway, 2007). Children were required to recall sequences of numbers in forwards (Digit Recall) and reverse order (Backwards Digit Recall), spatial locations on a 4 × 4 grid (Dot Matrix) and spatial locations at three points of a triangle whilst performing a secondary task, which required children to mentally rotate an object to identify whether it was the same or mirror image of another object (Spatial Span). These measures have good test–retest reliability (*r* = 0.64–84) in children (Alloway, Gathercole, & Pickering, 2006). There is high agreement between simple and backward/complex span tasks in children (*r* = 0.85 Alloway et al., 2006), suggesting that they measure the same underlying sub‐processes (see Unsworth & Engle, 2007). Therefore, performance on the four AWMA tasks was averaged to form a composite score of overall working memory ability, as in previous studies (Astle et al., 2015). The extent of near‐transfer to the individual tasks was also investigated in relation to its similarity with the training tasks, but these findings should be treated with some caution due to the larger number of comparisons. Greater transfer was expected on the Dot Matrix and Backwards Digit Recall tasks because they had similar structure and material to the Visual Data Link and Input Module training tasks, whereas the forwards Digit Recall and Spatial Span differed to the training tasks.

Academic achievement was assessed at baseline, immediate outcome and the 3 month outcome using the Reading Comprehension and Mathematical Reasoning subtests from the Wechsler Individual Achievement Test–II (Wechsler, 2005). The Reading Comprehension subtest includes questions that examine comprehension of written passages and sentences. It has excellent internal consistency for ages 9–14 (*r* = 0.94–96), excellent test–retest reliability (*r* = 0.93) and has reasonable convergent validity with other measures of reading achievement (*r* = 0.45–70). All responses were scored by the principal investigator (J.J.) to reduce subjective variability. The Mathematical Reasoning subtest predominantly includes single and multi‐step word problems relating to whole numbers, fractions or decimals, interpreting graphs, identifying patterns, rotating shapes and probability. It has excellent internal consistency for ages 9–14 (*r* = 0.92–95), excellent test–retest reliability (*r* = 0.94) and good convergent validity with other measures of Maths achievement (*r* = 0.59–67).

### 2.5 Data analyses

We analysed whether MetaCogmed and Cogmed significantly improved working memory, mathematical reasoning and reading comprehension relative to the Control group, and whether the MetaCogmed group improved significantly more relative to the Cogmed group. All analyses were intention‐to‐treat based, treating missing data as missing at least at random assumption. Baseline score adjusted analysis of covariance (ANCOVA) models were developed to compare group differences at the immediate and 3‐month outcomes. Further, multi‐level mixed models were developed to examine group differences in the time‐related change from baseline to immediate outcome and baseline to 3‐month outcome. The ANCOVA models included data on a complete case basis, whereas the mixed models also included data from participants with missing outcome data. The results of both analyses are reported for consistency. The ANCOVA results were preferred for the immediate effects because it is a common and recommended analysis method in randomized controlled trials with two time points (Van Breukelen, 2006). The mixed model results were preferred for the time‐related change at 3 months, where the additional follow‐up measurements added more power to the analyses compared to ANCOVA (Guo, Logan, Glueck, & Muller, 2013). All analyses were carried out using Stata statistical analytical software (StataCorp, 2017).

## 3 RESULTS

### 3.1 Baseline characteristics

Baseline data were collected for all participants at the point of randomization (*N* = 95). The number of data points (*N*), means, standard deviations (*SD*) and group differences (Δ) with 95% Confidence Intervals (CIs) are presented for all variables in Table 1. Between‐group differences were analysed using *t* tests for all continuous variables and chi‐square tests for gender. There were no significant differences across the three groups (all *p* > 0.05), suggesting that the randomization was effective. The only exception was for IQ, which was higher for the MetaCogmed group compared to the control group at borderline significance (*p* = 0.044). However, controlling for this factor did not significantly contribute to the regression models, and therefore, it was not added to the model results presented in the following sections.

**Table 1:** Baseline characteristics and tests of differences across groups

Variables	Control	MetaCogmed	Cogmed	Δ MetaCogmed versus Control (95% CI)	Δ Cogmed versus Control (95% CI)	Δ MetaCogmed versus Cogmed (95% CI)
Randomized (*N* = 95, 100%)	31 (33)	32 (34)	32 (34)	–	–	–
Gender: *N*	31	32	32	–	–	–
Male: *N*	16	18	16	–	–	–
Female: *N*	15	14	16	–	–	–
Other variables: (*N*)	31	32	32	–	–	–
Age: *M* (±*SD*)	12.52 (±0.96)	12.38 (±1.14)	12.63 (±1.25)	−0.14 (−0.67 to 0.39)	0.12 (−0.45 to 0.68)	−0.26 (−0.85 to 0.34)
IQ: * M* (±*SD*)	103.97 (±11.47)	109.81 (±10.74)	107.72 (±11.76)	5.84 (0.25 to 11.44)*	3.75 (−2.10 to 9.61)	2.09 (−3.53 to 7.72)
Primary outcome variables: (*N*)	31	32	32	–	–	
Maths: * M* (±*SD*)	101.52 (±9.31)	104.06 (±11.14)	104.09 (±11.62)	2.55 (−2.64 to 7.73)	2.58 (−2.74 to 7.89)	−0.03 (−5.72 to 5.66)
Reading: * M* (±*SD*)	101.65 (±9.14)	106.59 (±10.51)	103.81 (±11.93)	4.95 (−0.02 to 9.92)	2.17 (−3.20 to 7.54)	2.78 (−2.84 to 8.40)
Working memory: * M* (±*SD*)	103.46 (±9.82)	104.89 (±9.22)	105.12 (±9.11)	1.43 (−3.37 to 6.22)	1.66 (−3.11 to 6.43)	−0.23 (−4.81 to 4.35)
Near‐transfer variables: (*N*)	31	32	32	–	–	–
Digit recall (±*SD*)	100.81 (±11.69)	102.64 (±14.53)	100.88 (±13.35)	1.83 (−4.83 to 8.49)	0.07 (−6.25 to 6.40)	1.76 (−5.22 to 8.73)
Back digit (±*SD*)	101.24 (±14.27)	101.91 (±15.06)	107.64 (±14.03)	0.67 (−6.72 to 8.06)	6.40 (−0.73 to 13.52)	−5.73 (−13.00 to 1.55)
Dot matrix (±*SD*)	103.27 (±12.18)	103.98 (±14.24)	103.66 (±11.17)	0.72 (−5.97 to 7.40)	0.39 (−5.49 to 6.28)	0.33 (−6.07 to 6.72)
Spatial span (±*SD*)	108.53 (±16.93)	111.02 (±11.85)	108.29 (±14.90)	2.49 (−4.85 to 9.83)	−0.23 (−8.26 to 7.79)	2.72 (−4.00 to 9.45)

* *p* < 0.05.

### 3.2 Training adherence

Across the whole sample of 95 children, there were no significant differences (all *p* > 0.05) in the number of training sessions completed by the MetaCogmed (*M* = 17.8, *SD* = 5.42), Cogmed (*M* = 16.03, *SD* = 6.98) or Control groups (*M* = 18.15, *SD* = 5.83). Eighteen children withdrew during training and no further data were collected at the immediate or 3 month outcomes. This included three children from the Control group, six from the MetaCogmed group and nine from the Cogmed group. Drop‐out did not significantly differ across the groups, χ^2^(2, *N* = 95) = 3.49, *p* = 0.175. A total of 77 children completed the training programme: 28 children in the Control group, 26 in the MetaCogmed group and 23 in the Cogmed group (see Figure 1 for flow diagram). Outcome data were collected for all 77 children at the immediate and 3 month assessments. The majority of these children completed at least 20 training sessions (90.9%, *M* = 20.03, *SD* = 0.76) within 6 weeks (92.2%, *M* = 4.99, *SD* = 0.68), and there were no significant group differences in the number of training sessions completed (all *p* > 0.05): MetaCogmed (*M* = 20.12, *SD* = 0.91), Cogmed (*M* = 19.96, *SD* = 0.21) and Control (*M* = 20, *SD* = 0.9).

### 3.3 Immediate outcomes

**Figure 2:**
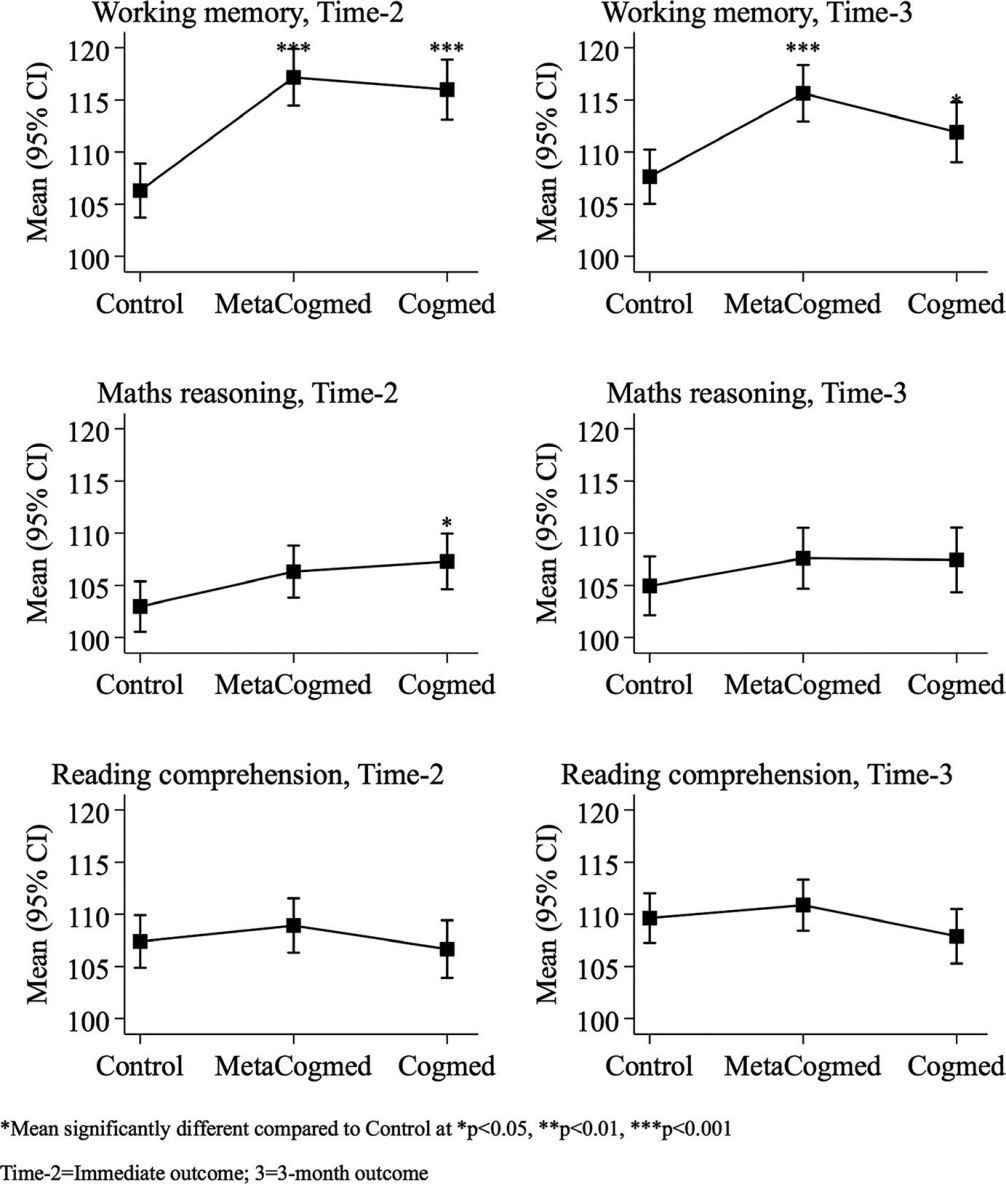
Baseline‐adjusted group means at immediate and 3 month outcomes (analyses of covariances)

**Figure 3:**
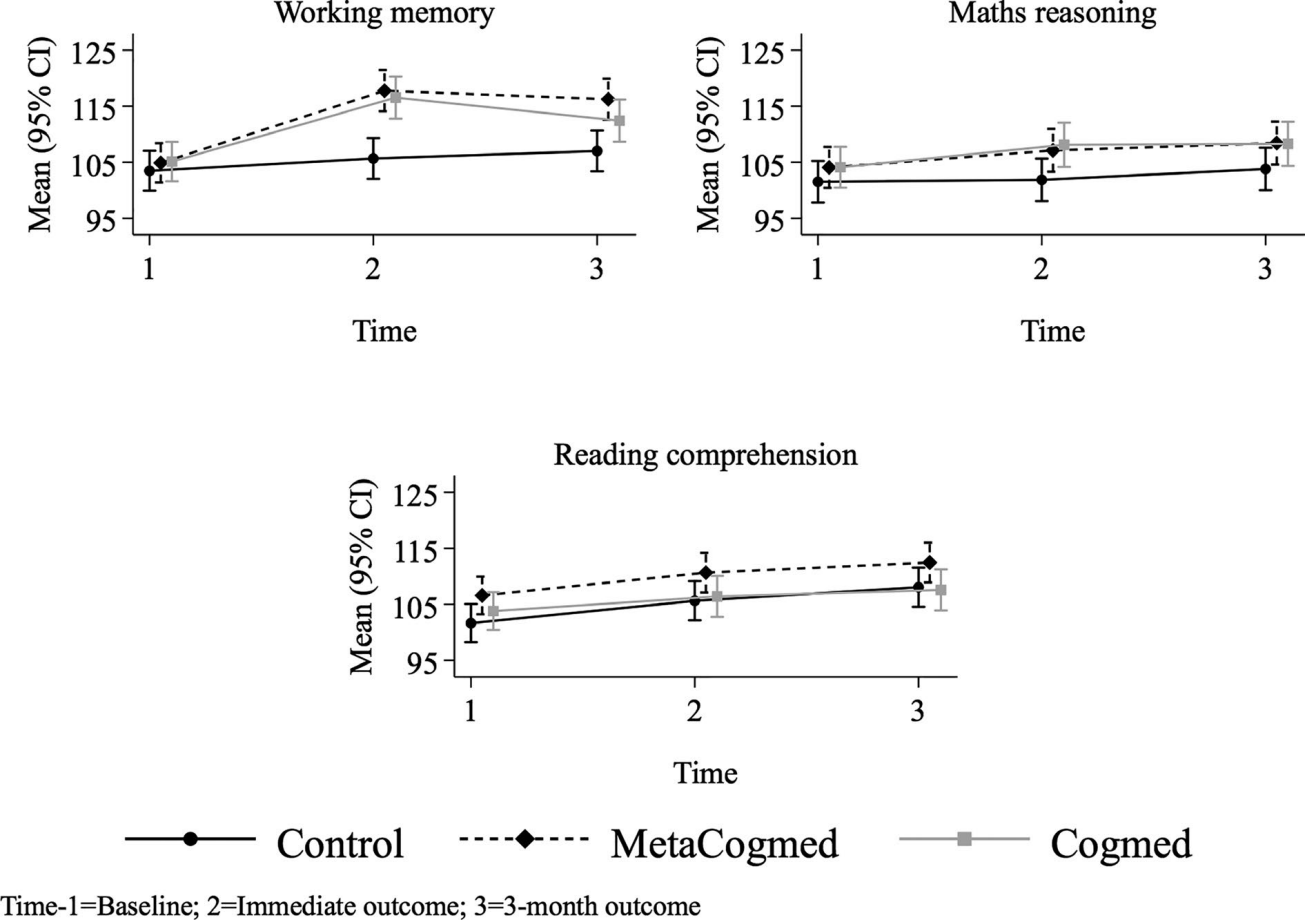
Plots of estimated means and 95% CIs (mixed models)

The results of the analyses of covariance (ANCOVAs), baseline adjusted group means, 95% CIs and mean differences (Δ) at the immediate and 3‐month outcome are presented in Table 2. The adjusted means and CIs for each group are plotted in Figure 2. Compared to the Control group, scores on the AWMA were significantly higher in the MetaCogmed (*p* < 0.001) and Cogmed groups (*p* < 0.001) at the immediate outcome. Average scores on Mathematical Reasoning were higher for the MetaCogmed and Cogmed groups compared to the Control group at immediate outcome. This difference was statistically significant for the Cogmed group (*p* = 0.019) and borderline significant for the MetaCogmed group (*p* = 0.059). There were no significant group differences in Reading Comprehension at the immediate outcome (all *p* > 0.05). The multi‐level models produced very similar results (see Table 3 and Figure 3), although the effect of Cogmed on Mathematical Reasoning was borderline significant (*p* = 0.068).

**Table 2:** Results of the analyses of covariance of primary near‐ and far‐transfer outcomes

Outcome variables	Time^a^	*N*	Control	MetaCogmed	Cogmed	Δ MetaCogmed versus Control	Δ Cogmed versus Control	Δ MetaCogmed versus Cogmed
			*M* (95% CI)	*M* (95% CI)	*M* (95% CI)			
Working memory	2	77	106.30 (103.70 to 108.90)	117.17 (114.46 to 119.87)	116.00 (113.12 to 118.87)	10.86 (7.11 to 14.61)***	9.69 (5.82 to 13.57)***	1.17 (−2.78 to 5.12)
	3	77	107.64 (105.04 to 110.24)	115.64 (112.94 to 118.34)	111.91 (109.03 to 114.78)	8.00 (4.25 to 11.75)***	4.27 (0.39 to 8.14)*	3.73 (−0.22 to 7.68)
Maths	2	77	102.96 (100.55 to 105.38)	106.32 (103.81 to 108.82)	107.29 (104.63 to 109.96)	3.35 (−0.13 to 6.83)	4.33 (0.73 to 7.93)*	−0.98 (−4.63 to 2.68)
	3	77	104.94 (102.13 to 107.76)	107.60 (104.68 to 110.52)	107.43 (104.33 to 110.54)	2.66 (−1.40 to 6.71)	2.49 (−1.70 to 6.68)	0.17 (−4.10 to 4.43)
Reading	2	77	107.39 (104.88 to 109.91)	108.92 (106.32 to 111.53)	106.66 (103.89 to 109.42)	1.53 (−2.10 to 5.16)	−0.74 (−4.48 to 3.01)	2.26 (−1.53 to 6.06)
	3	77	109.63 (107.26 to 112.00)	110.88 (108.42 to 113.34)	107.89 (105.28 to 110.50)	1.25 (−2.18 to 4.67)	−1.74 (−5.27 to 1.79)	2.99 (−0.59 to 6.57)

a Time: 2 = immediate, 3 = 3 month.

* *p* < 0.05.

*** *p* < 0.001.

**Table 3:** Results from the primary mixed models linear random intercept regressions

Variables	AWMA: coefficient (CI)	Maths: coefficient (CI)	Reading: coefficient (CI)
Time: (ref: baseline‐Time‐1)	–	–	–
Time‐2^a^	2.21 (−0.22 to 4.64)	0.34 (−2.33 to 3.01)	4.01 (1.33 to 6.66)**
Time‐3	3.55 (1.12 to 5.98)**	2.27 (−0.40 to 4.94)	6.43 (3.76 to 9.09)***
Group (ref: control)	–	–	–
MetaCogmed	1.43 (−3.58 to 6.44)	2.55 (−2.67 to 7.76)	4.95 (0.15 to 9.74)*
Cogmed	1.66 (−3.35 to 6.67)	2.58 (−2.64 to 7.79)	2.17 (−2.63 to 6.96)
Interaction (ref: baseline × control)	–	–	–
Time‐2 × MetaCogmed	10.66 (7.17 to 14.15)***	2.71 (−1.12 to 6.55)	0.07 (−3.76 to 3.91)
Time‐2 × Cogmed	9.22 (5.62 to 12.81)***	3.68 (−0.27 to 7.63)	−1.40 (−5.34 to 2.55)
Time‐3 × MetaCogmed	7.82 (4.33 to 11.31)***	2.09 (−1.74 to 5.93)	−0.55 (−4.38 to 3.28)
Time‐3 × Cogmed	3.76 (0.17 to 7.36)*	1.92 (−2.03 to 5.87)	−2.65 (−6.59 to 1.29)
Interaction (ref: baseline × Cogmed)			
Time‐2 × MetaCogmed	1.45 (−2.21 to 5.10)	−0.97 (−4.97 to 3.04)	1.47 (−2.53 to 5.47)
Time‐3 × MetaCogmed	4.06 (0.40 to 7.71)*	0.17 (−3.84 to 4.18)	2.10 (−1.90 to 6.10)

a Time: 2 = immediate, 3 = 3 month.

* *p* < 0.05.

** *p* < 0.01.

*** *p* < 0.001.

To investigate possible mechanisms of working memory training, the extent of near‐transfer was examined by comparing performance on the individual working memory tasks between the groups. If children only learn highly specific skills and strategies, then Cogmed may only improve performance on the Dot Matrix and Backwards Digit Recall tasks, which are structurally similar to the training tasks. The results of the ANCOVAs, baseline adjusted group means, 95% CIs and mean differences (Δ) are presented in Table 4. At the immediate outcome, the MetaCogmed group had significantly higher scores on the Dot Matrix (*p* = 0.009), Digit Recall (*p* = 0.001), Backwards Digit Recall (*p* < 0.001), and Spatial Span tasks (*p* = 0.006) compared to the Control group. Similarly, the Cogmed group had significantly higher scores on the Dot Matrix (*p* = 0.001), Digit Recall (*p* < 0.001) and Backwards Digit Recall tasks (*p* = 0.001), but not the Spatial Span task (*p* = 0.682) compared to the Control group. The MetaCogmed group also performed significantly higher on the Spatial Span task compared to the Cogmed group at the immediate outcome (*p* = 0.027). The multi‐level models produced very similar results (see Table 5); although performance on the Spatial Span task did not significantly differ between the MetaCogmed and Cogmed groups (*p* = 0.103).

**Table 4:** The results of the analyses of covariance on the individual near‐transfer task outcomes

Outcome variables	Time^a^	*N*	Control	MetaCogmed	Cogmed	Δ MetaCogmed versus Control	Δ Cogmed versus Control	Δ MetaCogmed versus Cogmed
			*M* (95% CI)	*M* (95% CI)	*M* (95% CI)			
Dot matrix	2	77	108.27 (103 to 114)	118.97 (113 to 125)	122.11 (116 to 128)	10.71 (3 to 19)**	13.85 (6 to 22)**	−3.14 (−12 to 5)
	3	77	114.54 (108 to 121)	117.15 (111 to 124)	114.25 (107 to 121)	2.61 (−6 to 11)	−0.29 (−9 to 9)	2.90 (−6 to 12)
Back digit	2	77	102.22 (98 to 106)	116.91 (113 to 121)	112.51 (108 to 117)	14.69 (9 to 21)***	10.29 (4 to 16)**	4.40 (−2 to 11)
	3	77	102.53 (98 to 107)	117.72 (113 to 122)	108.82 (104 to 114)	15.19 (9 to 22)***	6.29 (0 to 13)	8.90 (2 to 16)**
Spatial span	2	77	113.27 (109 to 118)	121.89 (118 to 126)	114.59 (110 to 119)	8.62 (3 to 15)**	1.32 (−5 to 8)	7.30 (1 to 14)*
	3	77	111.37 (107 to 116)	117.97 (113 to 123)	116.72 (112 to 122)	6.60 (0 to 13)	5.34 (−2 to 12)	1.26 (−6 to 8)
Digit recall	2	77	101.98 (98 to 106)	111.41 (107 to 115)	113.55 (109 to 118)	9.43 (4 to 15)***	11.57 (6 to 17)***	−2.14 (−8 to 4)
	3	77	102.17 (99 to 106)	109.92 (106 to 113)	107.52 (104 to 111)	7.75 (3 to 13)**	5.35 (0 to 10)*	2.40 (−3 to 8)

a Time: 2 = immediate, 3 = 3 month.

* *p* < 0.05.

** *p* < 0.01.

*** *p* < 0.001.

**Table 5:** Mixed model results for outcomes on the individual near‐transfer tasks

Variables	Dot Matrix: coefficient (CI)	Back Digit: coefficient (CI)	Spatial Span: coefficient (CI)	Digit Recall: coefficient (CI)
Time: (ref: baseline‐Time‐1)	–	–	–	–
Time‐2^a^	4.52 (−1 to 10)	0.02 (−4 to 4)	4.47 (0 to 9)	0.56 (−3 to 4)
Time‐3	10.8 (5 to 16)***	0.58 (−4 to 5)	2.78 (−2 to 8)	0.76 (−3 to 4)
Group: (ref: control)	–	–	–	–
MetaCogmed	0.72 (−7 to 8)	0.67 (−6 to 8)	2.49 (−5 to 10)	1.83 (−5 to 9)
Cogmed	0.39 (−7 to 8)	6.4 (−1 to 14)	−0.23 (−7 to 7)	0.07 (−7 to 7)
Interaction: (ref: baseline × control)	–	–	–	–
Time‐2 × MetaCogmed	10.7 (3 to 19)**	14.47 (8 to 21)***	7.65 (1 to 14)*	9.4 (5 to 14)***
Time‐2 × Cogmed	13.18 (5 to 22)**	8.54 (2 to 15)**	1.74 (−5 to 9)	11.36 (6 to 16)***
Time‐3 × MetaCogmed	2.66 (−5 to 11)	14.97 (9 to 21)***	5.54 (−1 to 12)	7.69 (3 to 13)**
Time‐3 × Cogmed	−1.03 (−9 to 7)	3.69 (−3 to 10)	5.2 (−2 to 12)	5.15 (0 to 10)*
Interaction: (ref: Baseline × Cogmed)				
Time‐2 × MetaCogmed	−2.48 (−11 to 6)	5.92 (−1 to 12)	5.91 (−1 to 13)	−1.97 (−7 to 3)
Time‐3 × MetaCogmed	3.69 (−5 to 12)	11.28 (5 to 18)***	0.33 (−7 to 7)	2.53 (−3 to 8)

a Time: 2 = immediate, 3 = 3 month.

* *p* < 0.05.

** *p* < 0.01.

*** *p* < 0.001.

### 3.4 Three month outcomes

Random intercept models were developed for each of the outcome variables to account for the variance due to repeated measures. The log likelihood ratio tests revealed that all models were highly significant compared to a single level model (Working Memory: χ^2^ = 83.34, *p* < 0.001; Maths: χ^2^ = 131.12, *p* < 0.001; Reading: χ^2^ = 91.03, *p* < 0.001), indicating a substantial amount of variance attributable at the individual/upper level. The resulting coefficients and 95% CIs from the random intercept models are presented in Table 3. The estimated means and CIs for each group are plotted in Figure 3.

The mixed models indicated that scores on the AWMA increased significantly more in the MetaCogmed (*p* < 0.001) and Cogmed groups (*p* = 0.040) compared to the Control group at the 3‐month outcome. Furthermore, improvements on the AWMA were significantly greater in the MetaCogmed group compared to the Cogmed group at the 3 month outcome (*p* = 0.030). Relative to the Control group, there was no significant improvement in Maths scores for the MetaCogmed (*p* = 0.285) or Cogmed groups (*p* = 0.340) at the 3‐month outcome. Similarly, there was no significant improvement in Reading scores for the MetaCogmed (*p* = 0.488) or Cogmed groups (*p* = 0.188) compared to the Control group at the 3‐month outcome. The ANCOVAs produced very similar results (see Table 2 and Figure 2), although the greater improvement in working memory performance in the MetaCogmed group compared to the Cogmed group was borderline significant (*p* = 0.06).

Concerning the extent of near‐transfer at 3 months, the coefficients and CIs from the mixed models are presented for each task in Table 5. At the 3‐month outcome, MetaCogmed significantly improved performance on the Digit Recall task compared to the Control group (*p* = 0.002) and on the Backwards Digit Recall task compared to the Control (*p* < 0.001) and Cogmed groups (*p* < 0.001). Cogmed only significantly improved performance on the Digit Recall task compared to the Control group (*p* = 0.045). The ANCOVAs confirmed these findings and revealed two additional effects that were borderline significant (see Table 4). This included the difference between the MetaCogmed and Control groups on the Spatial Span task (*p* = 0.055) and the difference between the Cogmed and Control groups on the Backwards Digit Recall task (*p* = 0.063).

## 4 DISCUSSION

We examined whether working memory training (Cogmed) and a novel combination of working memory and metacognitive strategy training (MetaCogmed) improved children's working memory and academic outcomes immediately and 3 months after training, relative to an adaptive control group. Overall, the results suggested that both interventions improved working memory performance at the immediate and 3‐month outcome. Furthermore, near‐transfer was greater in the MetaCogmed group compared to the Cogmed group at the 3‐month outcome. Regarding far‐transfer, both interventions improved mathematical reasoning relative to the control group, but this was only statistically significant for the Cogmed group and was not maintained at the 3‐month outcome. Neither intervention improved reading comprehension relative to the control group at either time point. Finally, there were no differences in mathematical reasoning or reading comprehension between the two working memory training groups. We discuss each of the main findings in turn.

### 4.1 Near‐transfer

The present study was the first to investigate and find near‐transfer from working memory training in children relative to an adaptive control group. For both the Cogmed and MetaCogmed groups, this improvement in working memory performance was observed not only at the immediate outcome but also at the 3‐month outcome, which indicates that the effect of working memory training can be long‐lasting. These findings extend upon previous developmental working memory training studies that have reported near‐transfer effects in comparison with a non‐adaptive or other active control group (Astle et al., 2015; Henry et al., 2014; Roberts et al., 2016), which may have been confounded by effects on expectancy and motivation (Shipstead et al., 2012). Furthermore, our results are broadly consistent with adult working memory training studies that have reported near‐transfer when compared to adaptive visual search training (Covey et al., 2019; Harrison et al., 2013) and adaptive general knowledge training (Anguera et al., 2012). The repeated and demanding activation of working memory at current capacity limits during training may have induced neuroplastic changes, affording increased capacity and/or led to the development of task‐specific strategies, automatization of basic processes and chunk learning that make more efficient use of capacity (von Bastian & Oberauer, 2014; Lövdén et al., 2010). These processes are not necessarily mutually exclusive and an important avenue for future research will be to determine to what extent training‐related improvements in working memory reflect increases in capacity, which maybe more likely to support far‐transfer (Lövdén et al., 2010).

We examined the extent and mechanisms of near‐transfer by analysing whether performance gains on the individual working memory tasks differed between the groups. As expected, Cogmed and MetaCogmed significantly improved performance on the Dot Matrix and Backwards Digit Recall tasks. These tasks are similar to multiple Cogmed training tasks and, therefore, may have afforded the same strategies (Dunning & Holmes, 2014). Cogmed and MetaCogmed also improved performance on the forwards Digit Recall task, showing generalization to a different task. Finally, compared to both the Control and Cogmed groups, only MetaCogmed improved performance on the Spatial Span task, which was the least similar to the Cogmed training tasks. These findings broadly correspond with recent meta‐analyses that have shown large or moderate near‐transfer on tasks that are very similar to the training and small, but significant, effects on less similar tasks (Aksayli, Sala, & Gobet, 2018; Melby‐Lervåg, Redick, & Hulme, 2016; Soveri, Antfolk, Karlsson, Salo, & Laine, 2017). Near‐transfer may depend on whether the training and transfer tasks share the same high‐order cognitive routine (Gathercole, Dunning, Holmes, & Norris, 2019); however, the contribution of improvements in capacity or more general cognitive resources is still a matter of debate (von Bastian & Oberauer, 2014).

### 4.2 Far‐transfer

To our knowledge, this is the first evidence that working memory training improves children's maths ability in the short term, compared to an active control group. This was statistically significant for the Cogmed group and borderline significant for the MetaCogmed group. Short‐term improvement in maths has previously been reported in a meta‐analysis of 11 working memory training studies in typically developing children (Sala & Gobet, 2017). However, this effect was not significant for the meta‐analysis of the six studies that used active control groups. This may be because expectancy and motivation were better controlled for, but it may reflect a lack of power due to the limited number of studies and the use of maths training as a control in two studies (Kuhn & Holling, 2014; Passolunghi & Costa, 2016). The only previous randomized controlled trial of Cogmed in typically developing children reported no improvement on a mixed assessment of maths ability (Hitchcock & Westwell, 2017). Although somewhat contrary to our findings, it may be possible that working memory training particularly improves mathematical reasoning, as a previous study showed specific improvements in word‐problem solving (Kuhn & Holling, 2014), which is more strongly associated with working memory capacity than other maths abilities (Peng et al., 2015). It is also important to acknowledge that training was provided in addition to school, as previous evidence indicates that replacing school lessons with working memory training leads to worse maths outcomes in the long term (Roberts et al., 2016).

Whilst our finding of far‐transfer to maths is promising and noteworthy, this effect was not maintained at the 3‐month outcome. This immediate effect may indicate that working memory training directly improved children's mathematical reasoning ability, rather than their capacity to learn and maths attainment, which would be more evident long term (Söderqvist & Bergman‐Nutley, 2015). Clearly for any intervention to be of important educational impact, it is necessary for the effect to be long‐lasting. A possible development for future research may be to investigate whether lower intensity “top up” training sessions delivered after the initial training are beneficial to maintain improvements in mathematical reasoning long term.

Although working memory training appears to be a promising method for improving mathematical reasoning ability, the academic benefits of Cogmed did not generalize to reading comprehension. The absence of an improvement in reading comprehension is consistent with the only other randomized controlled trial of Cogmed in typically developing children (Hitchcock & Westwell, 2017) and a meta‐analysis of 12 working memory training studies that found no benefits to reading, more generally (Sala & Gobet, 2017). Working memory training does not appear to benefit children's reading abilities, and our study suggests that this is the case even when there are large improvements in working memory performance.

### 4.3 Concurrent working memory and metacognitive strategy training

The comparison between MetaCogmed and Cogmed allowed us to examine whether metacognitive strategy training led to increased benefit relative to Cogmed training on its own. Importantly the placebo workbook controlled for possible non‐specific effects of metacognitive strategy training, allowing stronger inferences to be made than previous investigations (Partanen et al., 2015). The metacognitive booklet was shown to be effective to some extent because MetaCogmed was associated with greater near‐transfer, relative to Cogmed. This was the case for performance on the Spatial Span task at the immediate outcome, notably the task least similar to Cogmed, and average performance across the four working memory tasks at the 3‐month outcome. Metacognitive training may have increased children's awareness of which strategies were most effective, resulting in broader transfer to the Spatial Span task and better consolidation of these strategies for retrieval at the 3 month outcome. Previously, working memory and metacognitive strategy training has been shown to improve children's working memory immediately (Carretti et al., 2014) and 6 months after training (Partanen et al., 2015), compared to reading comprehension practice or education as usual. However, this is the first time that working memory and metacognitive strategy training has been shown to have an additional near‐transfer effect in children, compared to working memory training alone.

Although MetaCogmed led to immediate improvements in mathematical reasoning relative to the control group, metacognitive strategy training did not provide any additional benefits to maths or reading comprehension compared to Cogmed alone. This may be because Cogmed did not increase working memory capacity per se, or because the metacognitive training was ineffective at promoting generalization of the newly acquired capacity to the maths and reading tasks. This finding was consistent with a previous study in children with special educational needs (Partanen et al., 2015). Although working memory and metacognitive training have previously been shown to improve children's reading comprehension compared to reading comprehension practice (Carretti et al., 2014), this may have been because children were also taught specific reading strategies (Higgins et al., 2016; National Reading Panel, 2000). Working memory and metacognitive training interventions are still in their infancy and unlikely to be optimal without further research and development; yet MetaCogmed shows promise at maintaining improvements in working memory long term.

### 4.4 Strengths, limitations, and future directions

MetaCogmed is a standard, inexpensive, and timely intervention that can be delivered to a whole classroom by a single teacher and would be feasible for schools to implement, if it was found to be effective. However, the metacognitive intervention may have been too short and the exercises may have been too narrow to facilitate generalizable improvements across different domains, compared to existing interventions (Adey & Shayer, 1993). Incorporating teacher guidance, more diverse tasks, and interactive group work within our metacognitive workbook over a more extended time period may have greater potential to foster metacognition and facilitate far‐transfer.

A major strength and improvement upon much of the extant literature was the use of an adaptive control group and placebo workbook to control for the effects of expectancy and motivation. Adaptive visual search training has previously been shown to be an excellent control for subjective expectations in young adults (De Simoni & von Bastian, 2018). We also found no significant differences in drop‐out and completed training sessions, suggesting that engagement was similar across the groups. Whilst these are objective measures, they are also a fairly indirect indicator of engagement. One could potentially argue that they could have been supplemented with self‐report measures; however, children's self‐reports can be inaccurate (Goodman, Hinden, & Khandelwal, 2000) and may be limited by immature introspection and metacognition (Lyons & Zelazo, 2011).

One limitation of our study was that we were unable to ascertain whether the metacognitive workbook was effective at fostering metacognitive awareness and strategies. This may be achieved in future by utilizing existing self‐report (Schraw & Dennison, 1994), parent‐report (Gioia & Isquith, 2011) or task‐based measures (Krasny‐Pacini et al., 2015) of metacognition. More general limitations include the relatively small sample size compared to some previous investigations (Roberts et al., 2016) and considerable drop‐out, although this is comparable to other working memory training studies with children (Chacko et al., 2014; Hitchcock & Westwell, 2017).

## 5 CONCLUSION

The present study revealed that working memory training increases working memory performance in typically developing children both immediately and after a 3‐month delay. Short‐term improvements in mathematical reasoning highlight the potential educational impact of working memory training when provided in addition to school as usual. However, future work will need to examine whether these improvements can be maintained longer term and whether they generalize to other academic outcomes. Metacognitive strategy training in addition to working memory training facilitated greater near‐transfer 3 months after training relative to working memory training alone, suggesting that it may be an effective addition to cognitive skill‐based training programmes to maintain long‐term effects. Nevertheless, to fully realize its potential, children may require more practice applying metacognitive strategies to maths and reading exercises, and more diverse and interactive exercises to promote generalizable improvements in academic outcomes.

## CONFLICT OF INTEREST

The authors declare that there are no conflicts of interest.

## DATA AVAILABILITY STATEMENT

All materials and data are publicly available (https://osf.io/kxyf3).
