# Supplementary material for: The academic outcomes of working memory and metacognitive strategy training in children: A double‐blind randomized controlled trial
Source: Dev Sci. 2019 Jun 27;23(4):e12870. doi: 10.1111/desc.12870 (PMC7379186; doi:10.1111/desc.12870)
Supplement: Supplementary file 1 [file DESC-23-e12870-s001.pdf]

6. Want to think metacognitively? Then PME!

We previously explored the idea of ‘metacognition’, which is thinking about your thinking.

In this section we will be exploring how to think ‘metacognitively’ about carrying out a task. There are 3 key thinking skills: **P**lanning, **M**onitoring and **E**valuating.

Planning is your thinking before you start the task. This helps you to organise your thoughts and the steps you will take to complete the task.

Monitoring is your thinking when you are doing the task. This helps you to keep track of your thinking, your understanding and your progress.

Evaluating is your thinking after you have finished the task. This helps you to check that you have completed your goal and to think about how you completed it.

The Important Steps of PME

Study the diagram below. It shows you the questions you should ask yourself before, during and after a task.

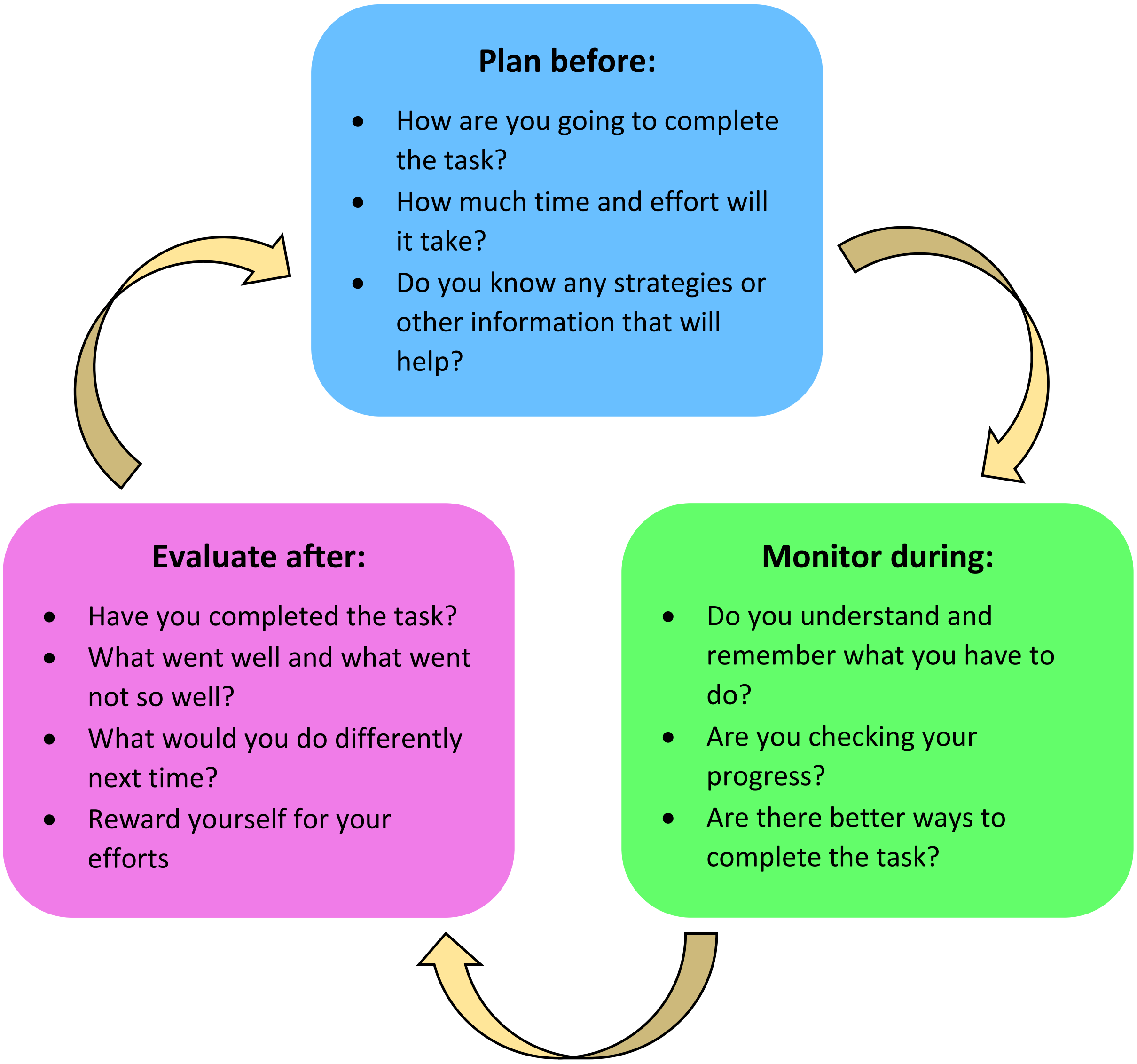

Exercise

In this exercise you need to read each thought and decide whether it is involved in planning, monitoring or evaluating. Draw a line from each box to connect them.

- **Example:** I am aware of my thinking
- I will use a strategy
- I took the right steps to solve the problem
- I can be proud of the effort I put in
- I know that this maths problem requires addition
- I don't think I am making good progress
- I need to think of another way to solve this problem
- Next time, I could plan things better
- I think this will be difficult, so I will spend a lot of time on it
- I understood the text and answered all the questions correctly
- I am checking that I understand and remember my goal
- I could have solved the problem in an easier way
- I will read the story first, then decide how to answer the questions

Plan

Monitor

Evaluate

Why use PME?

Thinking metacognitively will improve your performance on a range of different tasks. To do this, you need to form plans, check your progress and evaluate your performance.

Ultimately, this will help you to meet your goal more easily and more quickly. At first, it will take more time to learn how to think this way, but it will pay off in the future.

Think of it as an investment.

Summary

- Metacognition involves planning, monitoring and evaluating
- It is important to plan how you will complete a task, monitor your progress and evaluate your performance

10. PME in Practice – Memory 1

In section 2 you reflected on the 'Data Link' task that you have been training on. In this section we are going to put what we have learnt about PME into practice when you are completing the memory training on the computer.

Memory Exercise

In this exercise, we are going to focus on the 'Data Link' task that you have been doing in training. On the next few pages you will see questions that will ask you to think about the different parts of the 'Data Link' task.

By answering the questions, you will:

1. Plan how you will approach the task on page 32
2. Monitor your progress at the same time as completing the task on page 33
3. Evaluate your performance when you have finished the task on pages 33-34

It is very important that you follow this order.

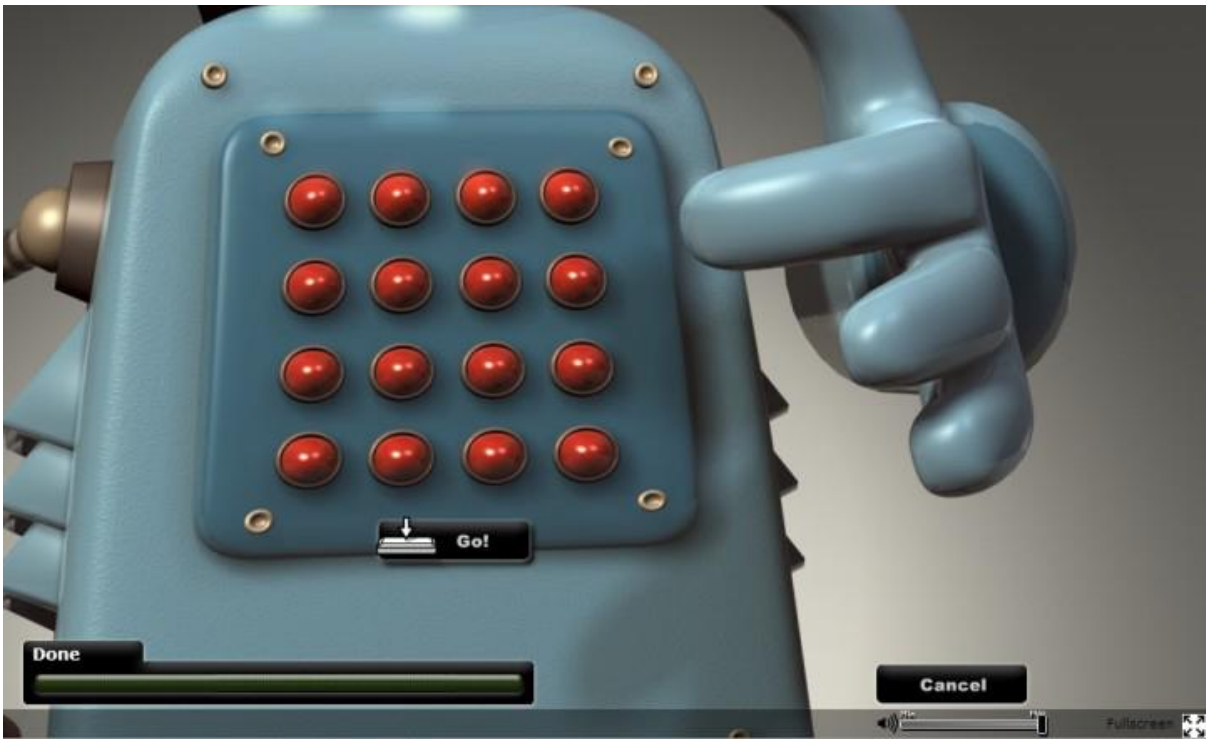

Plan – Complete before the task

Goals

What is your goal in the Data Link task? Be specific.

Difficulty

What level are you on? _______ How difficult is it going to be?

Very Easy	Easy	Neither	Hard	Very Hard
1	2	3	4	5

How long will it take you to complete? ________ minutes

Progress

What steps do you need to take to complete the Data Link task?

Strategies

Can you think of any strategies that will help you to remember? When will you use them?

Mind-set

How will you achieve a good mind-set to engage with the memory task?

Monitor – Complete during the task

Goals

Are you remembering your goals? **Yes / No**

Progress and Strategies

Are you following your plan? **Yes / No**

Are your strategies working well? **Yes / No**

Have you changed your plan or strategies? How?

Mind-set

Are you in the right mind-set to complete the task to the best of your ability? **Yes / No**

Evaluate – Complete after the task

Goals

Have you completed your goals? **Yes / No**

How well do you think you did on the Data Link task?

Very Poorly	Poorly	Neither	Well	Very Well
1	2	3	4	5

Progress & Strategies

How much of your plan did you follow?

None	Some	About Half	Most	All
1	2	3	4	5

When you were doing the Data Link task, what went well? Think about the steps you took and the strategies that you used.

What went not so well?

What would you do differently next time?

Mind-set

How motivated were you?

Very Unmotivated	Unmotivated	Neither	Motivated	Very Motivated
1	2	3	4	5

How focused were you?

Very Unfocused	Unfocused	Neither	Focused	Very Focused
1	2	3	4	5

What could you do next time to improve your motivation and focus?

Excellent Work! You have finished the workbook section now you just need to finish the training on the computer.

11. PME in Practice – Reading Comprehension 1

In section 3, you reflected on your reading comprehension. In this section we are going to practice PME on a reading comprehension task.

Reading Exercise

On the next few pages you will see questions that will ask you to think about the different parts of a reading task.

Now you will need to:

1. Briefly look at the reading task that you have to do on pages 38-39
2. Plan how you will approach the task on page 37
3. Monitor your progress whilst completing the task on page 38-39
4. Evaluate your performance when you have finished the task on pages 39-40

It is very important that you follow this order.

You must write your plan before you start reading the passage. You need to complete the monitoring section whilst you are reading and answering the questions. Once you have completed the questions, you need to complete the evaluation section.

Plan – Complete before the task

Goals

What is your goal in the Reading Exercise? Be specific.

Difficulty

How difficult will the reading task be?

Very Easy	Easy	Neither	Hard	Very Hard
1	2	3	4	5

How long will it take you to complete? _______ minutes

Strategies

What strategies will you use to help you understand and remember the passage? When will you use them?

Progress

What steps do you need to take to complete the Reading Exercise?

Mind-set

How will you achieve a good mind-set to engage with the reading task?

Classified Advertisements

A

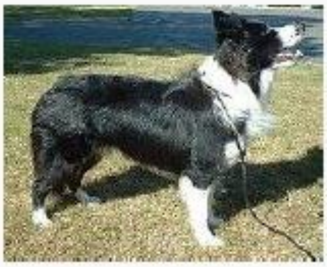

Sheepdog puppy

A beautiful, well bred male sheepdog puppy for sale.
We reluctantly have for sale a puppy dog. He is 10 months old and active so we would like him to go to a place in the country or to someone who can give him many walks.
Contact us @ jandksmith@yahoo.com or Telephone 01567 46578

B

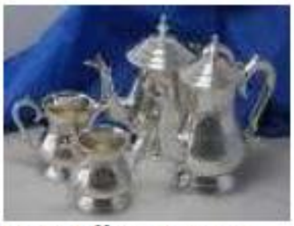

Vintage Silver tea set

This four piece tea service is available for sale. It has been used up to recently and is available with matching teaspoons. There are various other pieces of silver available. This tea set is for sale at High Holden showrooms, Oxfordshire Available Wednesdays or Saturdays. Price for this tea set is £50 or ono!
Contact :ebuy@hotmail.com or telephone 01564 687932

C

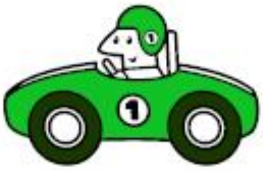

Austin green racing car 1997

Well preserved and loved racing car is for sale. It has been kept in the garage so it is in good condition and has 20,000 miles on the clock. One owner, now too old to enjoy his car, reluctantly wants a quick sale.
Contact him @ 01347 53218
Or at
Auto Motors 'Pen ride', Cumbria
Price between £2500 to £3000 considered.

D

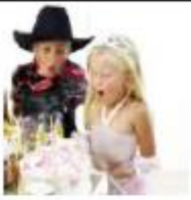

Want a fancy dress costume?

Come to The Theatre,
High Street, Helaston, Tyneside.
We have all sorts of costumes for sale from cowboys to vicars.
Costumes for young and old. Do not miss this one and only clearance sale.
Telephone 014876 891234 for appointment.
Hurry, costumes disappearing quickly.....!

Monitor – Complete during the task

Goals

Are you remembering your goals? **Yes / No**

Progress and Strategies

Are you checking that you understand the passage as you read? **Yes / No**

Are you following your plan? **Yes / No**

Are your strategies working well? **Yes / No**

Have you changed your plan or strategies? How?

Mind-set

Are you in the right mind-set to complete the task to the best of your ability? **Yes / No**

Reading Comprehension Questions

1) What kind of dog is for sale?

2) Does the owner want to sell him? What word illustrates this?

3) Is the tea set suitable to make tea in?

4) Why is the owner selling the car?

5) Does the theatre only have old fashioned costumes?

6) Is there usually a sale at this theatre?

Evaluate – Complete after the task

Goals

Have you completed your goal? **Yes / No**

Did you check your answers against the text? **Yes / No**

How many questions do you think you answered correctly? _____ / 6

Progress & Strategies

How much of your plan did you follow?

None	Some	About Half	Most	All
1	2	3	4	5

When you were doing the reading task, what went well? Think about the steps you took and the strategies that you used.

What went not so well?

What would you do differently next time?

Mind-set

How motivated were you?

Very Unmotivated	Unmotivated	Neither	Motivated	Very Motivated
1	2	3	4	5

How focused were you?

Very Unfocused	Unfocused	Neither	Focused	Very Focused
1	2	3	4	5

What could you do next time to improve your motivation and focus?

Well done! You have finished today's training!

12. PME in Practice – Maths Problem Solving 1

In section 4, you reflected on your maths problem solving. In this section we are going to practice PME on a maths problem solving task.

Maths Exercise

On the next few pages you will see questions that will ask you to think about the different parts of a maths task.

Now you will need to:

1. Read the question on page 43
2. Plan how you will approach the task on page 42
3. Monitor your progress whilst completing the task on page 43
4. Evaluate your performance when you have finished the task on pages 44-45

It is very important that you follow this order.

You must write your plan before you start reading the passage. You need to complete the monitoring section whilst you are reading and answering the questions. Once you have completed the questions, you need to complete the evaluation section.

Plan – Complete before the task

Goals

What is your goal in the Maths Exercise? Be specific.

Difficulty

How difficult is the Maths Exercise going to be?

Very Easy	Easy	Neither	Hard	Very Hard
1	2	3	4	5

How long will it take? ________ minutes

Progress

What steps do you need to take to solve the maths problem?

Strategies

What strategies could you use to help you solve the maths problem?

Mind-set

How will you achieve a good mind-set to engage with the maths task?

Spinner

A fair spinner has eight equal sections with different colours on each section. Two sections are blue, one is green, three are red, and two are yellow. What is the probability that I spin red or blue?

Show all your working.

Complete the plan section before answering the question.

Answer: ________

Monitor – Complete during the task

Goals

Are you remembering your goals? **Yes / No**

Progress and Strategies

Are you following your plan? **Yes / No**

Are your strategies working well? **Yes / No**

Have you changed your strategies? How?

Mind-set

Are you in the right mind-set to complete the task to the best of your ability? **Yes / No**

Evaluate – Complete after the task

Goals

Have you completed your goal? **Yes / No**

Did you check your answers? **Yes / No**

Progress & Strategies

How much of your plan did you follow?

None	Some	About Half	Most	All
1	2	3	4	5

When you were solving the maths problem, what went well? Think about the steps you took and the strategies that you used.

What went not so well?

What would you do differently next time?

Mind-set

How motivated were you?

Very Unmotivated	Unmotivated	Neither	Motivated	Very Motivated
1	2	3	4	5

How focused were you?

Very Unfocused	Unfocused	Neither	Focused	Very Focused
1	2	3	4	5

What could you do next time to improve your motivation and focus?

Congratulations! You have finished today's training!
