# Supplementary material for: The academic outcomes of working memory and metacognitive strategy training in children: A double‐blind randomized controlled trial
Source: Dev Sci. 2019 Jun 27;23(4):e12870. doi: 10.1111/desc.12870 (PMC7379186; doi:10.1111/desc.12870)
Supplement: Supplementary file 2 [file DESC-23-e12870-s002.pdf]

6. Attention

You may have noticed that the training requires you to pay close attention to the letters shown on screen. Attention is used to focus on important information so that we can use in some meaningful way. For example, in class you pay attention to what the teacher is saying because they are teaching you new information.

Word Exercise

In the word search below, try to find as many of the words related to memory as you can. The words can go in any direction. See how many you can cross off as you find them.

Attention

O	S	E	T	A	X	I	F	I	C	F	C	E	O
E	T	O	R	O	N	T	T	E	L	O	D	R	D
R	I	I	R	E	D	I	S	N	O	C	C	O	I
S	S	O	R	G	N	E	E	N	E	U	O	N	S
A	E	C	I	T	O	N	T	E	T	S	N	G	T
T	N	K	O	O	L	A	C	O	O	A	C	I	R
T	N	E	G	L	E	C	T	N	T	T	E	A	A
E	N	O	R	E	A	A	S	I	N	T	N	T	C
N	E	O	N	N	E	T	S	I	L	E	T	N	T
T	O	A	N	N	T	T	E	N	E	N	R	O	R
I	G	S	T	V	N	E	X	O	R	T	A	O	O
O	E	N	N	E	T	N	C	S	L	I	T	E	T
N	E	T	R	A	A	D	N	A	T	V	E	T	N
L	O	R	T	N	O	C	S	G	L	E	E	E	I

CONCENTRATE
LISTEN
LOOK
IGNORE
NEGLECT
ATTENTIVE
ATTEND
ATTENTION
CONTROL
FIXATE
FOCUS
ENGROSS
DISTRACT
NOTICE
CONSIDER

Play this puzzle online at : http://thewordsearch.com/puzzle/74616/

Summary

- The training requires you to pay close attention
- Attention is complex and there are many different types of attention

10. Training 3

In this section you are going to answer some questions on the training programme that you have been doing on the computer. Below is a picture of the training.

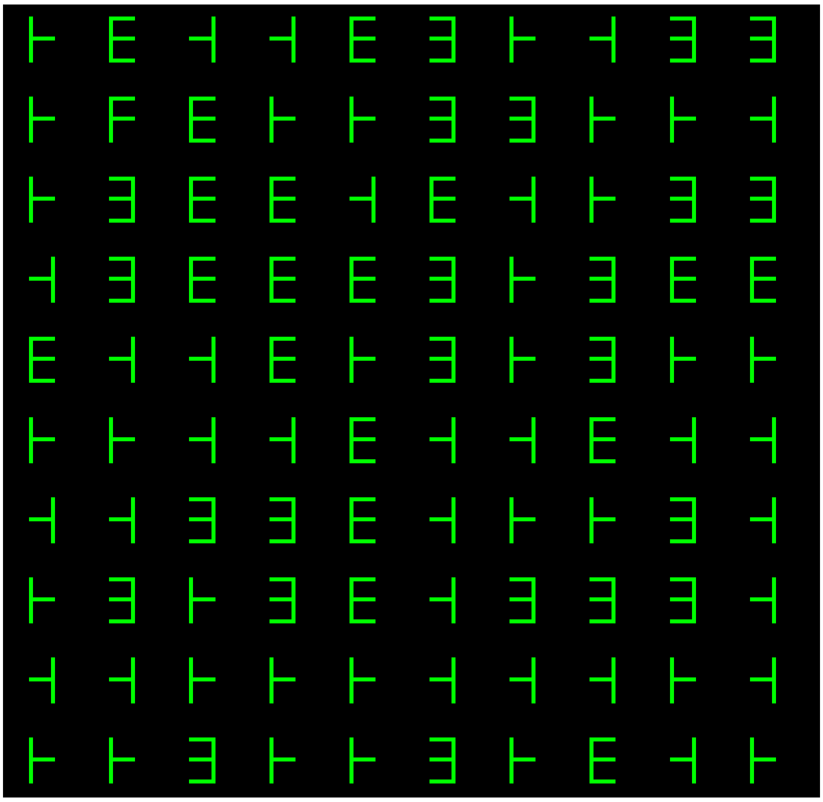

Exercise

In these questions, you need to say how much you agree with each statement by circling the appropriate number. If you disagree with the statement you will circle 1 or 2, if you agree with the statement you will circle 4 or 5. If you are not sure if you agree or disagree, then you will circle 3.

Think about each question carefully and then circle one number.

Example:

E) I enjoy playing sports.

Strongly Disagree	Disagree	Neither	Agree	Strongly Agree
1	2	3	(4)	5

1. I enjoy doing the training.

Strongly Disagree	Disagree	Neither	Agree	Strongly Agree
1	2	3	4	5

2. I think the training could be valuable to me.

Strongly Disagree	Disagree	Neither	Agree	Strongly Agree
1	2	3	4	5

3. I think the training programme is easy to use.

Strongly Disagree	Disagree	Neither	Agree	Strongly Agree
1	2	3	4	5

4. I am not trying very hard to do well on the training.

Strongly Disagree	Disagree	Neither	Agree	Strongly Agree
1	2	3	4	5

5. I think the training is important.

Strongly Disagree	Disagree	Neither	Agree	Strongly Agree
1	2	3	4	5

6. I think the training programme is difficult to use.

Strongly Disagree	Disagree	Neither	Agree	Strongly Agree
1	2	3	4	5

7. I would do this training programme again.

Strongly Disagree	Disagree	Neither	Agree	Strongly Agree
1	2	3	4	5

8. The training is fun to do.

Strongly Disagree	Disagree	Neither	Agree	Strongly Agree
1	2	3	4	5

9. I believe doing the training could be beneficial to me.

Strongly Disagree	Disagree	Neither	Agree	Strongly Agree
1	2	3	4	5

10. I am trying hard in the training.

Strongly Disagree	Disagree	Neither	Agree	Strongly Agree
1	2	3	4	5

11. I don't find the training very engaging.

Strongly Disagree	Disagree	Neither	Agree	Strongly Agree
1	2	3	4	5

12. I think the training is boring.

Strongly Disagree	Disagree	Neither	Agree	Strongly Agree
1	2	3	4	5

13. I put a lot of effort into the training.

Strongly Disagree	Disagree	Neither	Agree	Strongly Agree
1	2	3	4	5

14. I don't think the training is very important.

Strongly Disagree	Disagree	Neither	Agree	Strongly Agree
1	2	3	4	5

15. The training programme is very interesting.

Strongly Disagree	Disagree	Neither	Agree	Strongly Agree
1	2	3	4	5

16. It is important to me to do well on this training.

Strongly Disagree	Disagree	Neither	Agree	Strongly Agree
1	2	3	4	5

17. I find the training very challenging.

Strongly Disagree	Disagree	Neither	Agree	Strongly Agree
1	2	3	4	5

18. The training is very easy.

Strongly Disagree	Disagree	Neither	Agree	Strongly Agree
1	2	3	4	5

19. I find the training programme very engaging.

Strongly Disagree	Disagree	Neither	Agree	Strongly Agree
1	2	3	4	5

20. I am not putting much effort into the training.

Strongly Disagree	Disagree	Neither	Agree	Strongly Agree
1	2	3	4	5

Excellent Work! You have finished today's training.

11. Reading Comprehension 2

In this section you are going to practice some reading exercises. In the first exercise, you need to read the passage and answer some questions based on the information in the passage. In the second exercise, you need to find words that are related to the passage in a word search.

Reading Exercise

Classified Advertisements

A

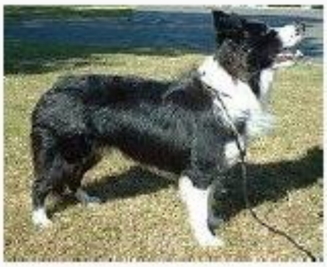

Sheepdog puppy

A beautiful, well bred male sheepdog puppy for sale.
We reluctantly have for sale a puppy dog. He is 10 months old and active so we would like him to go to a place in the country or to someone who can give him many walks.
Contact us @ jandksmith@yahoo.com or Telephone 01567 46578

B

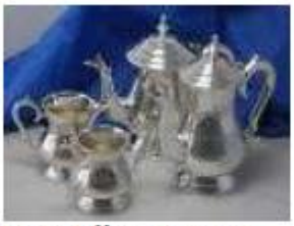

Vintage Silver tea set

This four piece tea service is available for sale. It has been used up to recently and is available with matching teaspoons. There are various other pieces of silver available. This tea set is for sale at High Holden showrooms, Oxfordshire Available Wednesdays or Saturdays. Price for this tea set is £50 or ono!
Contact :ebuy@hotmail.com or telephone 01564 687932

C

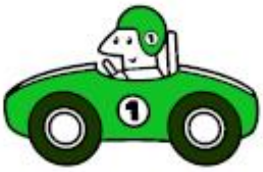

Austin green racing car 1997

Well preserved and loved racing car is for sale. It has been kept in the garage so it is in good condition and has 20,000 miles on the clock. One owner, now too old to enjoy his car, reluctantly wants a quick sale.
Contact him @ 01347 53218
Or at
Auto Motors 'Pen ride', Cumbria
Price between £2500 to £3000 considered.

D

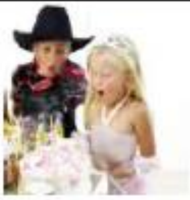

Want a fancy dress costume?

Come to The Theatre,
High Street, Helaston, Tyneside.
We have all sorts of costumes for sale from cowboys to vicars.
Costumes for young and old. Do not miss this one and only clearance sale.
Telephone 014876 891234 for appointment.
Hurry, costumes disappearing quickly…..!

Reading Comprehension Questions

1) What kind of dog is for sale?

2) Does the owner want to sell him? What word illustrates this?

3) Is the tea set suitable to make tea in?

4) Why is the owner selling the car?

5) Does the theatre only have old fashioned costumes?

6) Is there usually a sale at this theatre?

Word Exercise

In the word search below, try to find as many of the words related to the passage as you can. The words can go in any direction: to the right, left, up, down or diagonally. See how many you can cross off as you find them.

Classifieds

N	C	A	C	L	E	A	R	A	N	C	E	R	V
I	O	P	S	P	U	P	P	Y	V	O	E	H	I
S	N	P	S	C	L	A	S	S	I	F	I	E	D
N	S	O	L	P	G	O	D	P	E	E	H	S	A
I	I	I	R	C	R	W	L	A	C	T	I	V	E
C	D	N	E	Y	S	S	P	E	E	E	T	E	S
T	E	T	V	S	V	I	Y	D	S	A	D	H	T
C	R	M	L	O	A	S	E	F	S	O	E	N	A
S	E	E	I	V	H	L	D	O	N	P	O	P	E
S	D	N	S	I	I	L	E	M	U	T	S	O	C
R	I	T	M	O	O	R	W	O	H	S	N	Y	I
N	R	E	L	U	C	T	A	N	T	L	Y	E	N
A	U	S	T	I	N	O	S	S	A	E	A	C	P
E	G	A	T	N	I	V	N	T	S	A	O	U	Y

CLASSIFIED
AUSTIN
RELUCTANTLY
COSTUME
SALE
APPOINTMENT
SHOWROOM
VINTAGE
SHEEPDOG
SILVER
ACTIVE
CONSIDERED
CLEARANCE
PUPPY

Play this puzzle online at : http://thewordsearch.com/puzzle/89482/

Well done! You have finished today's training!

12. Maths Problem Solving 2

In this section you are going to practice some maths exercises. In the first exercise, you need to read the question and solve the maths problem. In the second exercise, you need to find numbers hidden amongst other numbers.

Maths Exercise

Spinner

A fair spinner has eight equal sections with different colours on each section. Two sections are blue, one is green, three are red, and two are yellow. What is the probability that I spin red or blue?

Show all your working.

Answer: ________

Number Exercise

In this exercise you need to search for the numbers shown in the list below. It can be written horizontally, vertically and diagonally! There are 30 in total, see how many you can find!

8	5	1	1	5	8	9	4	7	8	4	4	9	8	6
8	5	5	2	5	7	9	7	8	5	6	0	0	8	3
7	4	4	2	6	7	7	6	9	9	8	2	0	2	0
8	2	3	5	6	8	1	4	8	0	7	8	4	3	2
3	7	2	7	7	2	5	9	8	8	3	1	1	9	9
5	1	5	2	8	5	7	8	8	9	1	9	8	5	3
7	3	6	3	1	7	6	8	3	2	4	5	6	4	6
4	1	5	4	3	6	9	2	2	3	5	6	5	8	7
3	8	1	3	7	4	3	7	2	1	1	9	8	6	3
3	1	5	5	8	0	8	3	5	2	8	0	9	9	2
2	5	6	7	6	1	5	7	6	3	4	1	4	2	9
8	2	6	4	4	8	8	8	1	1	4	0	3	6	7
7	5	5	2	8	7	9	7	4	0	9	0	4	0	8
4	4	9	8	5	4	3	2	1	3	9	3	9	3	3
2	4	7	6	5	6	4	1	7	2	2	5	6	3	2

Number List

11568	26576	72221	88532
11589	36732	78432	89434
12257	45678	78498	90100
22114	48574	78721	97534
22356	56678	78777	98543
24546	58331	85457	98964
24564	69260	87533	
25632	69982	87974	

Congratulations! You have finished today's training!
